# Mutational spectrum of acquired resistance to reversible versus irreversible *EGFR* tyrosine kinase inhibitors

**DOI:** 10.1186/s12885-020-06920-3

**Published:** 2020-05-12

**Authors:** Svenja Wagener-Ryczek, Carina Heydt, Juliane Süptitz, Sebastian Michels, Markus Falk, Christina Alidousty, Jana Fassunke, Michaela Angelika Ihle, Markus Tiemann, Lukas Heukamp, Jürgen Wolf, Reinhard Büttner, Sabine Merkelbach-Bruse

**Affiliations:** 1 Institute of Pathology, University Hospital of Cologne, Kerpener Str. 62, D-50937 Cologne, Germany; 2 Department I of Internal Medicine, University Hospital of Cologne, Cologne, Germany; 3 Insitute for Haematopathology, Hamburg, Hamburg Germany; 4 NEO New Oncology GmbH, Cologne, Germany

**Keywords:** NSCLC, EGFR, TKI, Acquired resistance, Afatinib, Erlotinib, Gefitinib

## Abstract

**Background:**

Over the past years, EGFR tyrosine kinase inhibitors (TKI) revolutionized treatment response. 1st-generation (reversible) EGFR TKI and later the 2nd –generation irreversible EGFR TKI Afatinib were aimed to improve treatment response. Nevertheless, diverse resistance mechanisms develop within the first year of therapy. Here, we evaluate the prevalence of acquired resistance mechanisms towards reversible and irreversible EGFR TKI.

**Methods:**

Rebiopsies of patients after progression to EGFR TKI therapy (> 6 months) were targeted to histological and molecular analysis. Multiplexed targeted sequencing (NGS) was conducted to identify acquired resistance mutations (e.g. EGFR p.T790M). Further, Fluorescence in situ hybridisation (FISH) was applied to investigate the status of bypass mechanisms like, MET or HER2 amplification.

**Results:**

One hundred twenty-three rebiopsy samples of patients that underwent first-line EGFR TKI therapy (PFS ≥6 months) were histologically and molecularly profiled upon clinical progression. The *EGFR* p.T790M mutation is the major mechanism of acquired resistance in patients treated with reversible as well as irreversible EGFR TKI. Nevertheless a statistically significant difference for the acquisition of T790M mutation has been identified: 45% of afatinib- vs 65% of reversible EGFR TKI treated patients developed a T790M mutation (*p*-value 0.02). Progression free survival (PFS) was comparable in patients treated with irreversible *EGFR* irrespective of the sensitising primary mutation or the acquisition of p.T790M.

**Conclusions:**

The *EGFR* p.T790M mutation is the most prominent mechanism of resistance to reversible and irreversible EGFR TKI therapy. Nevertheless there is a statistically significant difference of p.T790M acquisition between the two types of TKI, which might be of importance for clinical therapy decision.

## Background

Lung cancer is one of the leading causes of cancer related deaths worldwide [1]. Administration of epidermal growth factor receptor (EGFR) tyrosine kinase inhibitors (TKI) to patients with activating mutations in the *EGFR* gene, especially exon 19 deletions and exon 21 p.L858R point mutations, has significantly improved treatment and outcome of advanced-stage lung cancer patients [2].

Five to 50% of patients with lung adenocarcinomas carry activating mutations within the *EGFR* gene with huge differences between geographical distribution and populations [3]. Activating mutations confer patients susceptible to treatment with EGFR-tyrosine kinase inhibitors (TKIs). Objective tumour shrinkage is reported in approximately 75% of patients [3]. Nevertheless, acquired resistance to TKIs and secondary progression is being observed after a median time of 8 to 14 months in nearly all patients [4].

Until the emergence of osimertinib, first-line therapies were mostly administered with reversible (gefitinib, erlotinib) [5, 6] or irreversible TKIs (afatinib) [7]. Molecular analyses revealed a limited number of different resistance mechanisms. The most frequent mechanism (50–60%) is the gate-keeper point mutation p.T790M which lowers affinity of first-line TKIs to the ATP binding pocket [8, 9]. Less frequent resistance mechanisms (5–15%) are the activation of bypass receptor tyrosine kinases, such as *ERBB2* and *MET* amplifications [10, 11]. Infrequently, mutations within the genes encoding the downstream signalling molecules BRAF, KRAS, PIK3CA and CTNNB1 are observed [4]. A completely different and poorly understood mechanism abolishing sensitivity towards EGFR TKI involves the histological transformation into small cell or sarcomatoid lung cancer phenotypes [12]. Also compound resistance by multiple mechanisms in the same or in different tumour locations have been encountered [13].

As different resistance mechanisms require precise diagnostics and elicit a wide portfolio of different and effective second-line therapies [14], we here addressed the question whether the frequencies of resistance mechanisms differ between first-line therapies with reversible and irreversible TKIs. So far the prevalence of the *EGFR* p.T790M mutation and other resistance mechanisms after treatment with reversible first-generation EGFR TKI was investigated in different studies with low patient numbers (*n* = 37) as well as larger cohorts (*n* = 155) [15]. Especially, the mutational spectrum of irreversible second-generation EGFR TKI afatinib was only investigated in studies with low patient numbers (*n* = 4, *n* = 20) [16, 17]. Moreover, these studies included patients with second-line EGFR-TKI treatment. Therefore we compiled diagnostic and follow-up data of two very large German pathology centres.

## Methods

### Study population

Patients included into this study were biopsied prior to primary treatment and were diagnosed with non-resectable non-small cell lung cancer revealing an activating *EGFR* mutation in exon 19 or 21 at two study sites (Institute for Pathology, University Hospital Cologne and Institute for Hematopathology, Hamburg). All patients received therapy with one of the first-generation TKIs gefitinib or erlotinib or the second-generation TKI afatinib for a minimum of 6 months duration and were rebiopsied after clinically evident secondary progression. Rebiopsies were evaluated for histology, presence of p.T790M, amplifications in *MET* or *ERBB2*, and mutations in *KRAS, BRAF, PIK3CA, PTEN* or *CTNNB1*. DNA extraction was done as described in detail previously [18].

All patients consented into treatment according to GCP regulations and into molecular diagnostics according to institutional practice. Procedures were approved by the local Ethics Committees.

### Targeted parallel sequencing

Multiplex PCR-based target enrichment was performed as described in detail previously [18, 19] using a customized lung cancer panel covering 14 lung cancer related genes. Isolated DNA was amplified with an Ion AmpliSeq Custom DNA Panel (Thermo Fisher Scientific, Waltham, MA, USA), and the Ion AmpliSeq Library Kit 2.0 (Thermo Fisher Scientific) according to the Ion AmpliSeq Library Preparation User Guide (Thermo Fisher Scientific). The panel comprises a subset of cancer relevant genes including: *AKT1*, *ALK, BRAF*, *CTNNB1*, *DDR2*, *EGFR*, *ERBB2*, *KRAS*, *MAP 2 K1*, *MET*, *NRAS*, *PIK3CA*, *PTEN* and *TP53*.

Depending on DNA concentration, DNA was alternatively amplified with an updated version of the above described panel, namely the GeneRead DNAseq Targeted Panel V2 (Qiagen, Hilden, Germany) and the GeneRead DNAseq Panel PCR Kit V2 (Qiagen) according to the GeneRead DNASeq Gene Panel Handbook (Qiagen) as described previously [20]. This panel covers three additional cancer related genes: *KEAP1*, *FGFR2* and *FGFR3* .

From both types of PCR products, libraries were constructed using the Gene Read DNA Library I Core Kit and the Gene Read DNA I Amp Kit (Qiagen, Hilden, Germany). After end-repair and adenylation, NEXTflex DNA Barcodes were ligated (Bio Scientific, Austin, TX, USA). Barcoded libraries were amplified and then the final library product was quantified with Qubit dsDNA HS Assay Kit (Thermo Fisher Scientific) on the Qubit 2.0 Fluorometer (Thermo Fisher Scientific), diluted and pooled in equal amounts. Finally, 12 pM of the constructed libraries were sequenced on the MiSeq (Illumina, San Diego, CA, USA) with a MiSeq reagent kit V2 (300-cycles) (Illumina) following the manufacturer’s recommendations.

Data were exported as FASTQ files. Alignment and annotation was done using a modified version of a previously described method (Peifer et al., 2012). BAM files were visualized in the Integrative Genomics Viewer (http://www.broadinstitute.org/igv/, Cambridge; USA). A 5% cut-off for variant calls was used and results were only interpreted if the coverage was > 200.

### Fluorescence in situ hybridisation analyses

Fluorescence in situ hybridisation (FISH) for *MET* and *ERBB2* amplifications were performed on formalin-fixed, paraffin-embedded tissue specimens using dual-colour labelled hybridization probes (ZytoLight SPEC MET/CEN7 Dual Color Probe and ZytoLightSPEC ERBB2/CEN 17 Dual Color Probe (ZytoVision)). Sections of 1.5 μm tumour material were cut and hybridized overnight with labelled probes for *MET* and *ERBB2* respectively. Review of fluorescence signals was performed at 630x magnification and scored according to defined guidelines [21];.

### Statistical analysis

The Qui-Square Test was used to calculate the differences in prevalence of T790M by the Chi Square Calculator of www.socscitastics.com. Statistical differences in duration until resistance acquisition under EGFR TKI therapy were calculated using the student’s t-test as given by www.socscitastics.com.

## Results

### Patient collection and molecular analysis

**Fig. 1:**
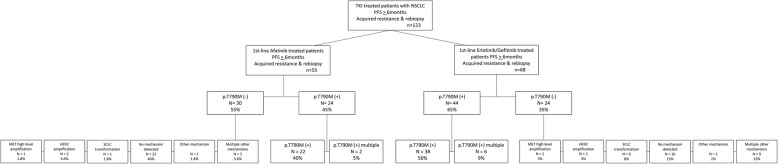
Flow Chart of patient collection and analysis of re-biopsies. 123 patients under EGFR TKI treatment with a PFS > 6 months and acquired resistance were rebiopsied (**a**). A cohort of 55 patients acquired resistance under irreversible (**b**) and 68 under reversible EGFR TKI therapy (**c**). Please note, that one afatinib-treated patient carried a primary EGFR Exon 19 duplication and was therefore excluded from the p.T790M prevalence calculation.

From January 2014 to January 2017, patients with primary sensitizing *EGFR* mutations were treated first-line with either afatinib, or reversible EGFR TKIs erlotinib/gefitinib respectively (Table 1). Progression free survival (PFS) or partial response for at least 6 months (PFS > 6 months) under EGFR TKI therapy was chosen as criteria to include only patients without pre-existing *EGFR* p.T790M mutations. 21% of EGFR-mutated and subsequently Afatinib-treated patients progressed in less than 6 month. Approximately 10% of EGFR-mutated and subsequently treated with either erlotinib or gefitinib progressed in less than 6 months. Those patients were not taken into the analysed cohort within this study as they were supposed to be primary resistant (e.g. because of pre-existent resistance clones.) At the time point of clinical progression, patients were re-biopsied for mutational profiling. Upon this, we analysed in total 123 patients, fulfilling the above criteria (Suppl. Table 1). 55 of those patients had been treated with afatinib and 68 patients had received gefitinib or erlotinib (Fig. 1). All patients carried an activating *EGFR* mutation before therapy, including 83 primary *EGFR* Exon 19 deletions, 39 primary *EGFR* p.L858R mutations as well as one other *EGFR* mutation (Exon 19 duplication). The patient, carrying the EGFR Exon 19 duplication was excluded from p.T790M prevalence testing. Noteworthy, the distribution of primary *EGFR* mutation types was balanced between the group of patients treated with a reversible and irreversible EGFR TKI, respectively. The most common initial mutation was the *EGFR* deletion p.E746_A750del, followed by the *EGFR* point mutation p.L858R and less common *EGFR* deletions in Exon 19, codons 752 to 759. Sensitivity of baseline biopsy analysis was set to 1% allelic fraction (1% AF). Nevertheless, we further ensured to excluded any possible patients with pre-existing p.T790M mutations via our study design with PFS > 6 months, as described above.

**Table 1:** Clinicopathological features of patients with NSCLC rebiopsied after EGFR TKI resistance acquisition

No. of cases	123
baseline morphology	NSCLC 100%
**EGFR TKI**	
afatinib	55
gefitinib	35
erlotinib	33
**median age (range)**	68 (40–87)
< 65 years (%)	24
> 65 years (%)	76
**sex (%)**	
male	53
female	70
**primary EGFR mutation**	
EGFR exon 19 deletion	89
EGFR exon 21 p.L858R	33
EGFR exon 19 duplication	1

Tumour material of EGFR TKI -resistant patients was examined for histological transformation and genetic mutations. All samples showed the histological subtype of adenocarcinoma, except in one tumour sample a small cell lung cancer phenotype was detected. All samples were analysed for genetic mutations within the described target genes to identify common resistance mechanisms besides *EGFR* p.T790M, such as *EGFR* p.S797S, *EGFR* Exon20 duplications and insertions. Further mutations, which may lead to resistance, were taken into account, i.e. activating *CTNNB-1* or *PIK3CA* mutations. Additionally, bypass mechanisms of resistance, which include *MET* high level amplifications, *ERBB2* amplifications were analysed using FISH. *MET* intermediate or low level amplifications (classification criteria as listed in the methods section) were not considered as a mechanism of resistance since their biological/therapeutical relevance is currently under discussion.

### Prevalence of acquired resistance mechanisms to first-line irreversible EGFR TKI afatinib

**Fig. 2:**
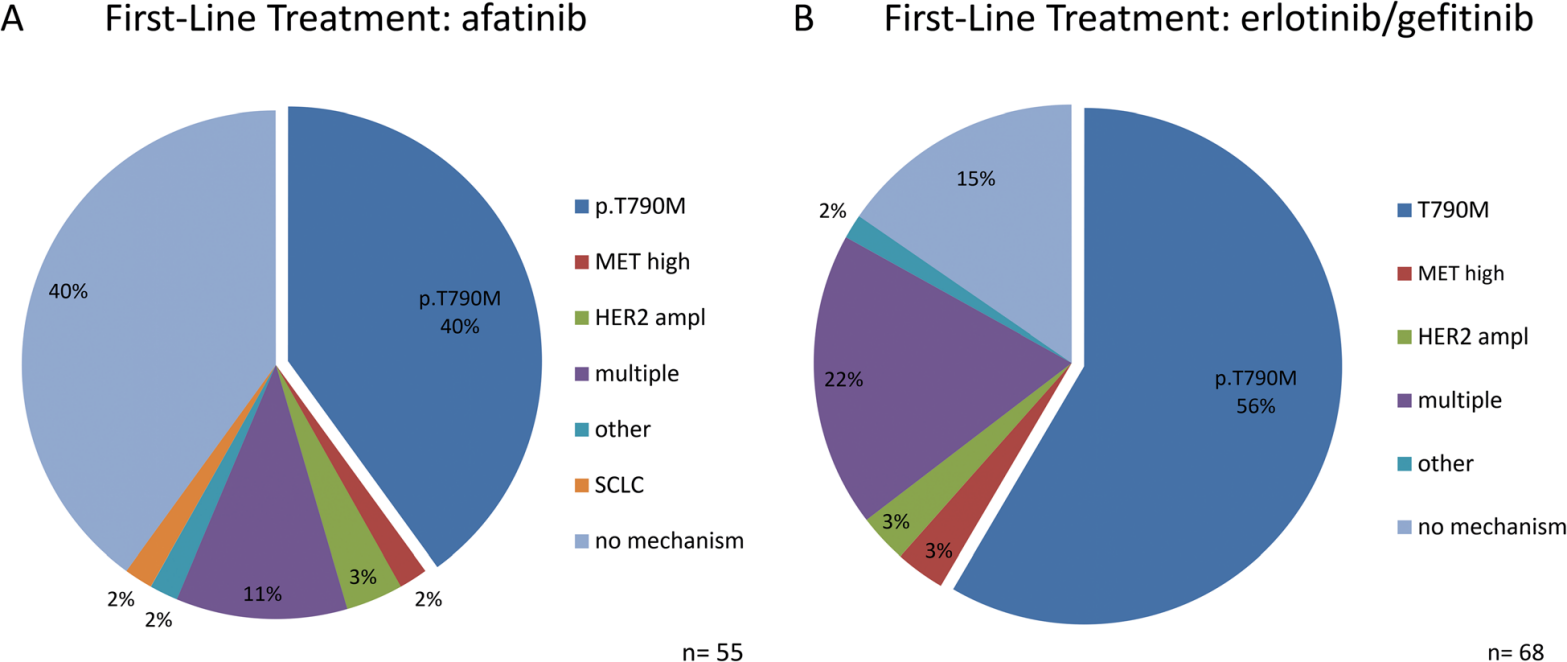
Spectrum of acquired resistance mechanisms. Frequencies of acquired resistance mechanisms under reversible (**a**) and irreversible (**b**) *EGFR* TKI therapy. The *EGFR* p. T790M gatekeeper mutation ist the major mechanism of resistance in reversible as well as irreversible *EGFR* TKI treated patients. Frequencies of alternative resistance mechanisms besides p.T790M are comparable. Note, section p.T790M covers only patients with T790M as exclusive resistance mechanism. Section of multiple resistance mechanisms includes patients with p.T790M plus *MET* or *HER2* amplification.

**Fig. 3:**
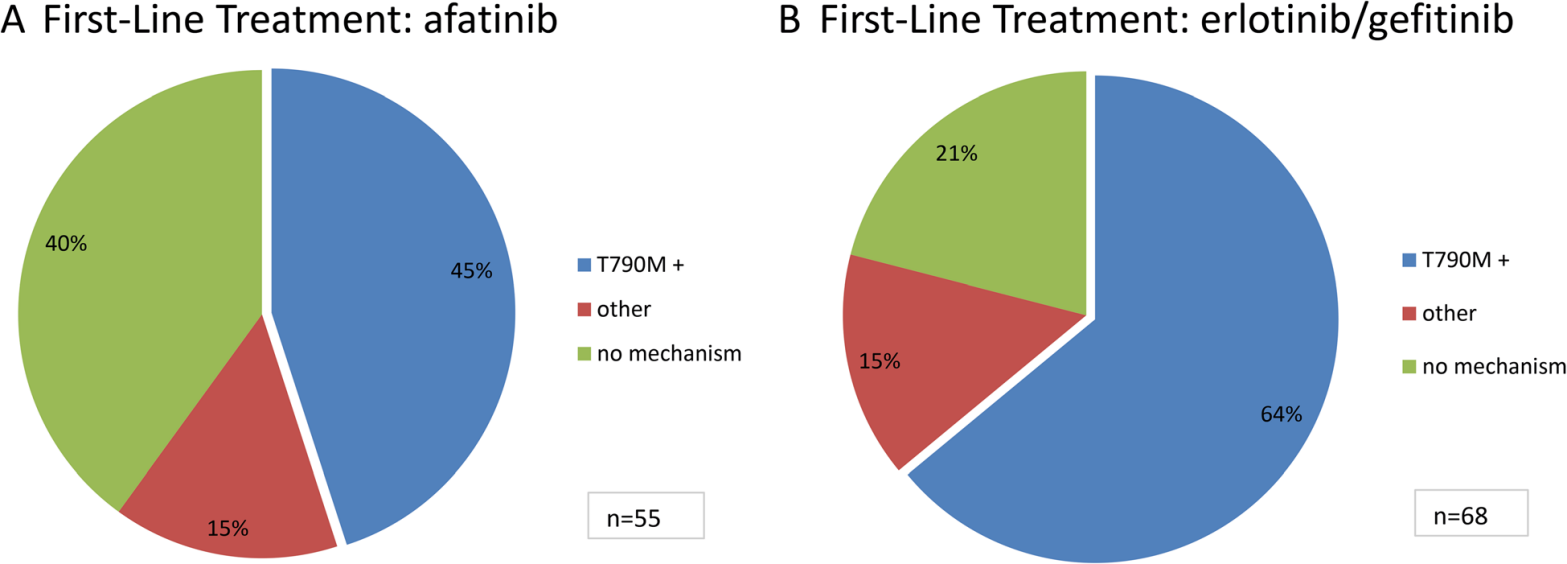
Frequency of p.T790M acquisition. Frequency of total p.T790M acquisition in patients under reversible (**a**) and irreversible (**b**) EGFR TKI therapy for > 6 months, respectively. Chi Square statistics identified a significant difference in the prevalence of p.T790M mutation in reversible vs irreversible EGF TKI treated patients (*p* = 0.019)

**Fig. 4:**
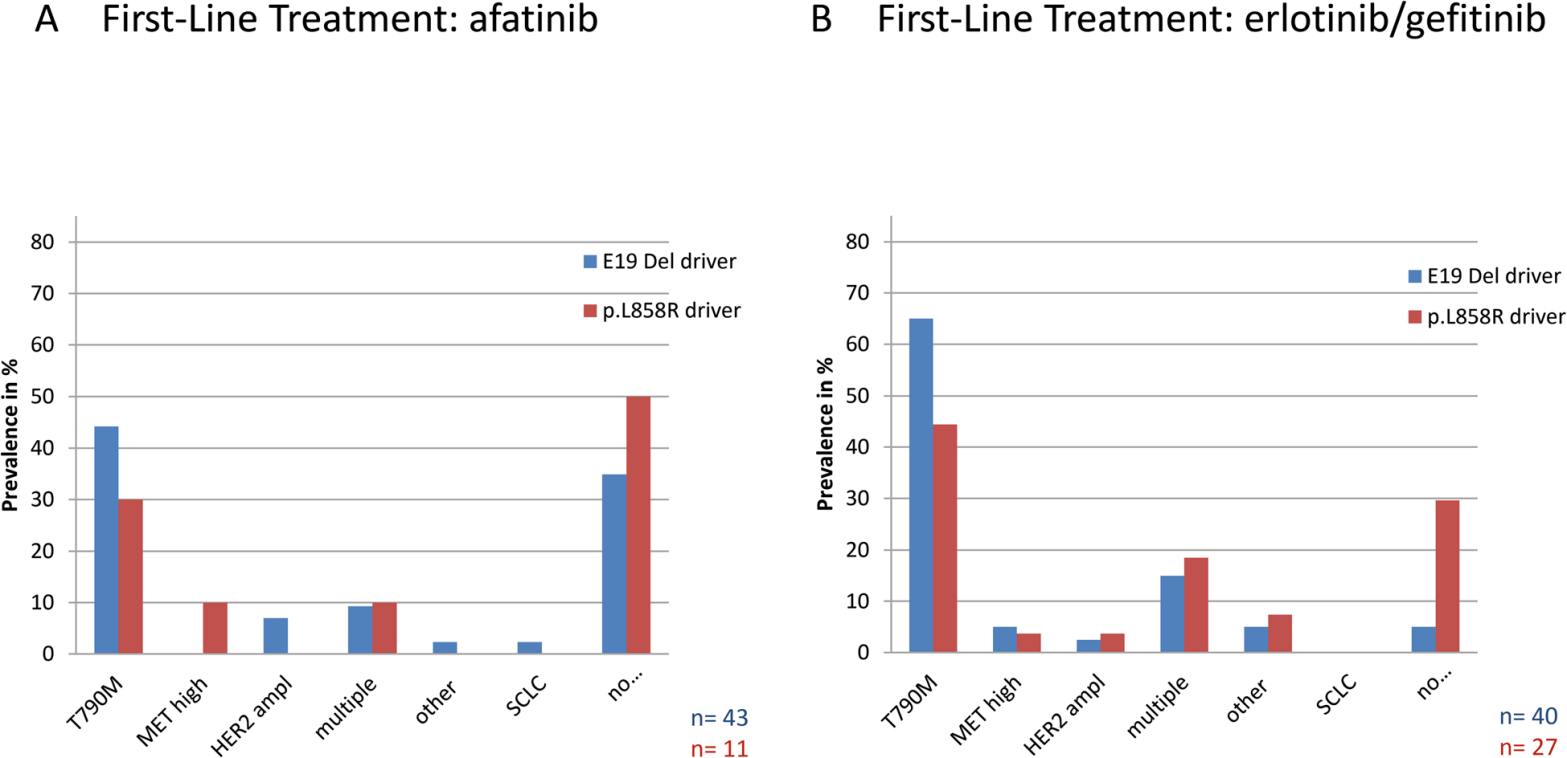
Spectrum of acquired resistance mechanisms in dependence on primary driver mutation. Frequencies of acquired resistance mechanisms under reversible (**a**) and irreversible (**b**) *EGFR* TKI therapy on the background of primary driver mutation. Note, section p.T790M covers only patients with T790M as exclusive resistance mechanism. Section of multiple resistance mechanisms includes patients with p.T790M plus *MET* or *HER2* amplification

**Fig. 5:**
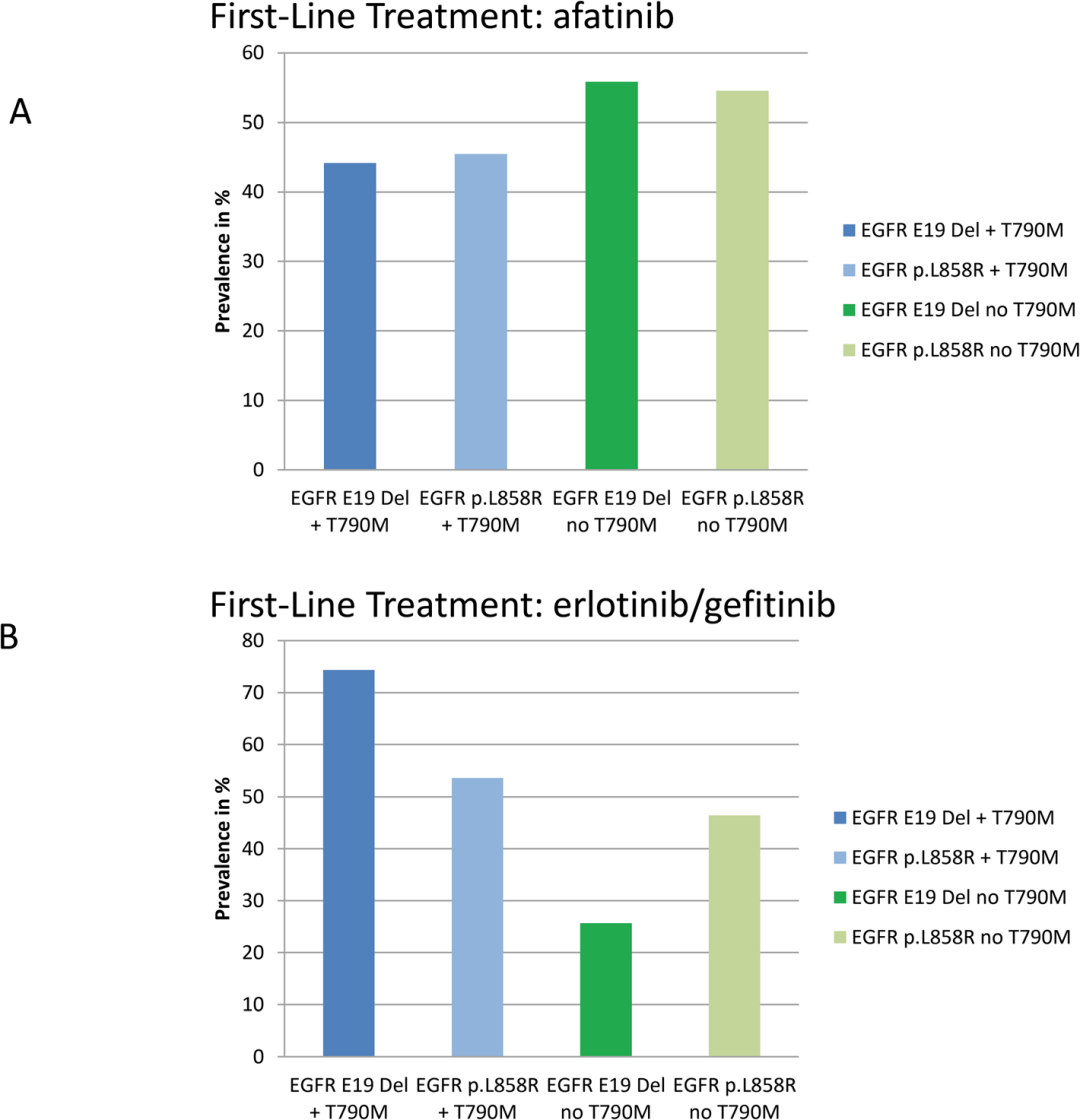
Frequency of p.T790M acquisition in dependence on primary driver mutation. Frequency of total p.T790M acquisition in patients under reversible (**a**) and irreversible (**b**) EGFR TKI therapy for > 6 months, respectively. Frequency of p.T790M acquisition in EGFR Exon 19 mutated patients differs significantly in reversible and irreversible EGFR TKI treated patients determined by Qui Square test (*p* = 0.005)

In 22 of 55 patients, the gatekeeper mutation *EGFR* p.T790M (40%) was the most prominent mechanism of resistance to afatinib (Fig. 2). Two additional patients showed supplementary to p.T790M, *MET* amplifications as additional resistance mechanism (Fig. 3). 19 of 43 (44%) patients with a primary *EGFR* Exon 19 deletion acquired an *EGFR* p.T790M mutation as resistance mechanism to afatinib. From 11 patients, carrying a primary *EGFR* p.L858R mutation, three (30%) developed the *EGFR* p.T790M gatekeeper mutation in response to afatinib therapy (Figs. 4 and 5).

Among patients without *EGFR* p.T790M mutation, one patient (1.8%) acquired resistance via a high level *MET* amplification. Two patients (3.6%) were determined to acquire resistance via amplification of *ERBB2*. Four of the patients (11%) showed multiple routes of acquired resistance, in detail *MET* high level plus *ERBB2* amplification and *MET* high level amplification plus *EGFR* p.T790M mutation. None of the cases showed an *EGFR* resistance mutation other than p.T790M in response to afatinib (Fig. 2). In addition, 22 patients (40%) did not show any known mechanism of resistance (Fig. 2). 4 patients (9%), carrying multiple resistance mechanisms had *EGFR* Exon 19 deletions prior to therapy, while only one patient (10%) showed multiple resistance mechanisms after p.L858R primary *EGFR* mutation. Fifteen patients (35%) with a primary *EGFR* Exon 19 deletion and five (50%) with a primary *EGFR* p.L858R mutation developed resistance to afatinib without any detectable mechanism (Fig. 4).

### Prevalence of acquired resistance mechanisms to first-line reversible EGFR TKI erlotinib and gefitinib

In 38 of 68 patients, the gatekeeper mutation *EGFR* p.T790M (56%) was the most prominent mechanism of resistance to first-generation EGFR TKI (erlotinib or gefitinib) (Fig. 2). Six additional patients showed supplementary to p.T790M *MET* or *ERBB2* amplifications as a further resistance mechanism (Fig. 3).

Among patients without *EGFR* p.T790M mutation, two patients (3%) acquired resistance via a high level *MET* amplification. Another two patients (3%) were determined to acquire resistance via amplification of *ERBB2*. Fifteen of the patients (22%) showed multiple routes of acquired resistance, in detail *MET* high level plus *ERBB2* amplification and *MET* high level amplification plus *EGFR* p.T790M mutation. None of the cases showed an *EGFR* resistance mutation other than p.T790M in response to reversible first-generation EGFR TKI (Fig. 2). In addition, 10 patients (15%) did not show any known mechanism of resistance (Fig. 2).

### Prevalence of acquired resistance mechanisms to first-line afatinib in comparison to reversible EGFR TKI therapy

Although the gatekeeper mutation *EGFR* p.T790M is the most prominent mechanism of resistance in both cohorts, the prevalence is significantly lower under afatinib therapy in comparison to first-generation EGFR TKI therapy. (45% vs. 65%, *p* = 0.02, see Fig. 3, Chi-Square Test). Frequencies of alternative resistance mechanisms are comparable between afatinib and first-generation EGFR TKI treated patients. Notably, the proportion of resistant patients with unknown mechanisms of resistance is higher after afatinib therapy (40% vs 15%, *p* = 0.001) Fig. 2). Regarding the prevalence of *EGFR* p.T790M in combination with either of the primary activating *EGFR* mutations, *EGFR* Exon 19-mutant tumours acquired an *EGFR* p.T790M mutation as resistance mechanism more frequently than tumours with *EGFR* exon 21 mutations after reversible first-generation EGFR TKI therapy (75 and 54%, respectively, *p* = 0.07). Contrary, patients with primary *EGFR* Exon 19 deletions and activating p.L858R mutations acquired an EGFR p.T790M mutation with a comparable prevalence after afatinib treatment (44 and 45%, *p* = 1, Chi Square Test) (Fig. 4).

### Median duration until progression and resistance acquisition under first-generation reversible EGFR therapy

**Fig. 6:**
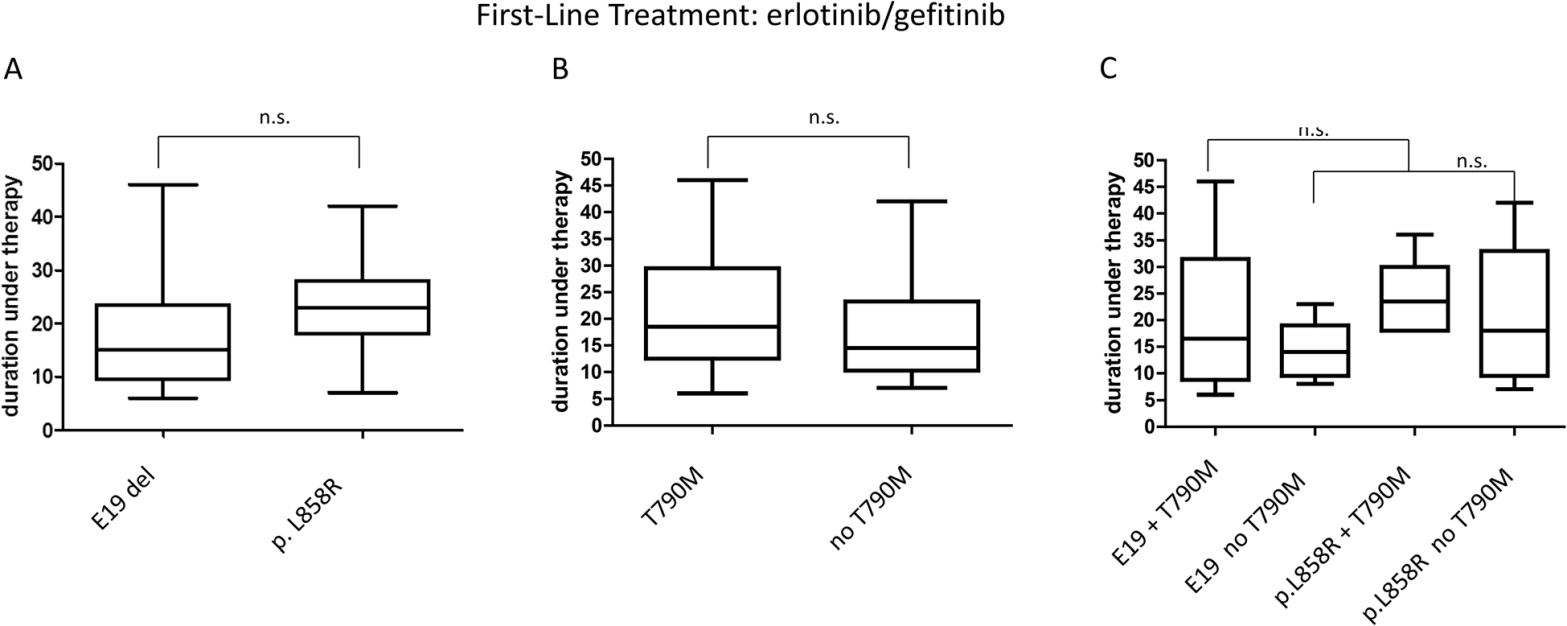
Duration until resistance acquisition under reversible EGFR TKI therapy. Duration until resistance acquisition under reversible EGFR TKI therapy in dependence on primary driver mutation (**a**) and p.T790M acquisistion status (**b**) respectively and combined (**c**). n.s. =no statistical significance (determined by student’s t-test)

Median period of first-generation EGFR TKI therapy until progression and resistance acquisition was 17 months regarding the total of 68 patients. Median duration until progression differed slightly in the presence of *EGFR* p.T790M mutation (17 vs 14 months) (Fig. 6 b). There was a difference in duration of response in regard to the primary mutation, nevertheless it was not found to be statistically significant. Patients with primary *EGFR* Exon 19 deletions showed a median of 15 month under therapy whereas *EGFR* p.L858R mutated patients showed a median of 24 months PFS (*p* = 0.2, Student’s t-test)(Fig. 6 a). Evaluating the PFS combining both types of driver mutation with the acquisition of p.T790M showed a median of 16 months in p.T790M mutated patients with a primary *EGFR* Exon 19 deletion in contrary to 23 months in patients with primary p.L858R (*p* = 0.3, Student’s t-test) (Fig. 6 a-c).

### Median duration until progression and resistance acquisition under afatinib therapy

**Fig. 7:**
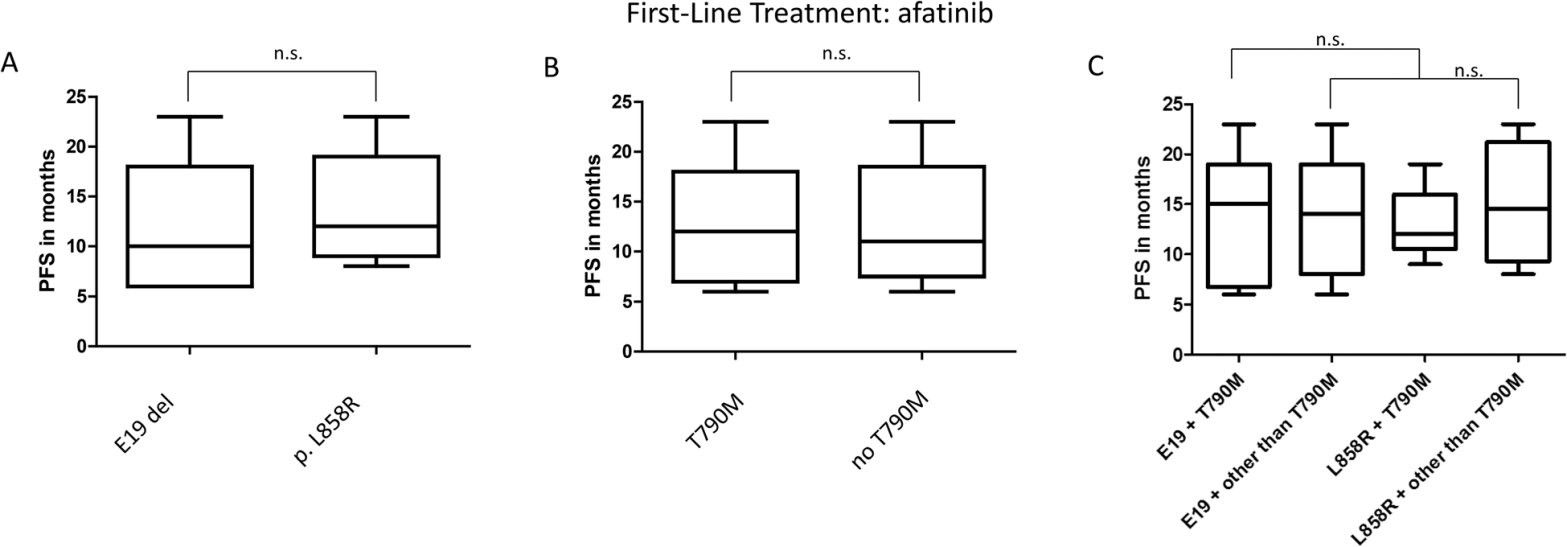
Duration until resistance acquisition under irreversible EGFR TKI therapy. Duration until resistance acquisition under irreversible EGFR TKI therapy in dependence on primary driver mutation (**a**) and p.T790M acquisistion status (**b**) respectively and combined (**c**). n.s. =no statistical significance (determined by student’s t-test)

Median period of afatinib therapy until progression and resistance acquisition was 12.6 months regarding the total of 55 patients. Median duration until progression did not differ significantly in the presence of *EGFR* p.T790M mutation (12 vs 11 months, *p* = 1, Student’s t-test) (Fig. 7 b). Specimen showing alternative mechanisms of resistance also had a PFS of 11.8 months. There was no statistical difference in duration of response in regard to the primary mutation. Patients with primary *EGFR* Exon 19 deletions as well as *EGFR* p.L858R mutated patients showed a median of 13 months PFS (Fig. 7 a). Evaluating the PFS combining both types of driver mutation with the acquisition of p.T790M showed a median of 15 months PFS in p.T790M mutated patients with a primary *EGFR* Exon 19 deletion in contrary to 11 months in patients with primary p.L858R (Fig. 7 a-c). Nevertheless, there was no statistically significant difference between both groups.

**Fig. 8:**
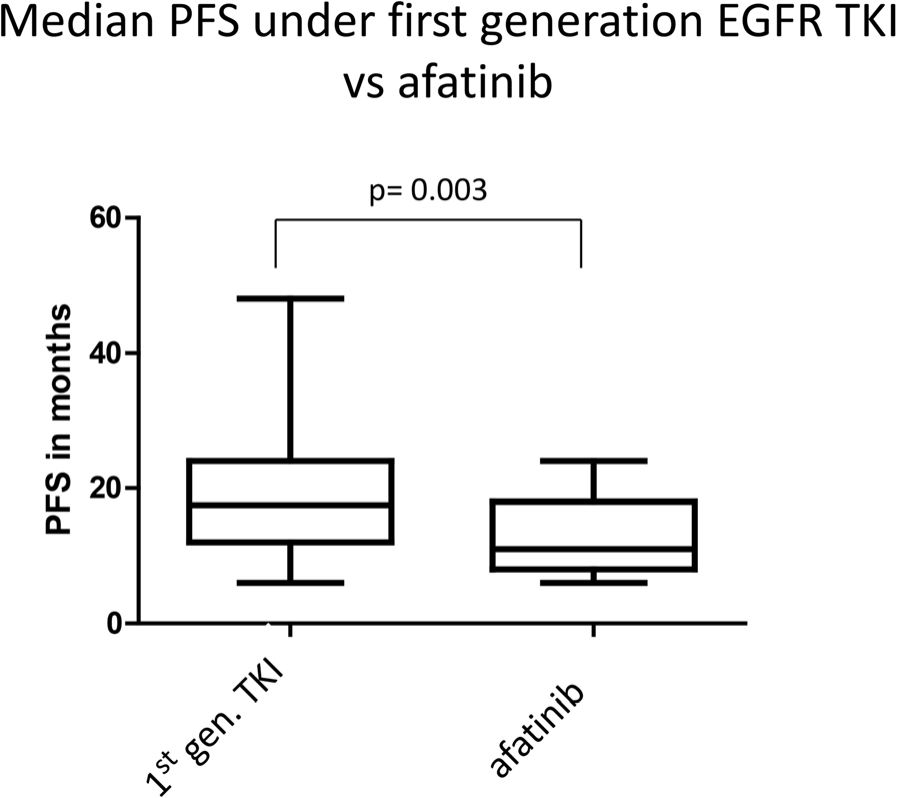
Duration until resistance acquisition under reversible and irreversible EGFR TKI therapy. Median PFS until resistance acquisition under reversible (first generation EGFR TKI erlotinib and gefitinib) an irreversible EGFR TKI (afatinib) therapy. Statistical significance is indicated by *p* = 0.003 (determined by student’s t-test)

Comparing median PFS under reversible first-generation and irreversible EGFR TKI shows a significant difference in duration with 21 months versus 13 months (*p* = 0.003) (Fig. 8).

## Discussion

The present retrospective study confirmed that the major mechanism of resistance to afatinib treatment is the *EGFR* p.T790M gatekeeper mutation. Nevertheless, the resistance mutation was detected with a lower prevalence than for reversible EGFR TKIs (erlotinib and gefitinib), 45% vs 64% *p* = 0.02. This is in contrast to previous reports by Wu et al., who showed a similar prevalence for *EGFR* p.T790M after afatinib or reversible EGFR TKI treatment (both 50–60%, *n* = 14,first-line afatinib). Due to the relatively small patient population of first-line afatinib-treated patients with *n* = 14, Wu et al. could not detect a statistically significant difference in the prevalence of EGFR p.T790M acquisition between reversible and irreversible EGFR TKI (*p* = 0.83) ( [16]). Two further prospective studies by Campo et al. and Tanaka et al. confirmed the lower prevalence of p.T790M in irreversible EGFR TKI treated patients. Campo et al. detected the EGFR p.T790M in 36% of afatinib treated patients (*n* = 11) [22]. Tanaka and co-workers deciphered the acquisition of p.T790M in 43% of afatinib treated patients (*n* = 37) [17]. Nevertheless, both studies present small cohorts of afatinib treated patients, which might be too low to draw significant conclusions from. However, our study presents the same numeric trend of a lower prevalence of p.T790M in irreversible EGFR TKI treated patients, which was determined to be statistically significant. Still, *EGFR* p.T790M mutation is the most prominent mechanism of resistance in afatinib treated patients. These findings imply that afatinib-treated patients should equally benefit from treatment with third-generation *EGFR* TKIs, like osimertinib, and need to be screened for emergence of the p.T790M resistance mutation. These novel emerging inhibitors are specific for the *EGFR* p.T790M mutated isoform of the EGFR receptor [23, 24]. According to El Kadi and coworkers, the formation of EGFR T790M mutation is initiated by AICDA-mediated deamination of the 5-methylcytosine following therapy with either of the EGFR TKI. Nevertheless they observed differential gene expression of AICDA under different treatment conditions (type and dose of EGFR TKI) [25]. Therefore, a different frequency of T790M acquisition under reversible and irreversible EGFR TKI is conceivable. Moreover the rate of residual growing cancer cells under/after EGFR TKI therapy that can support AICDA- mediated deamination may differ between various TKI, thereby leading to different frequencies of acquired T790M.

Small cell lung cancer transformation has been reported as an alternative mechanism of resistance to first-generation *EGFR* TKI in 3–14% of *EGFR* TKI-treated patients [15, 26]. This transformation arises upon TKI blockade of *EGFR* signalling in combination with additional mutations, such as inactivation of *RB1*. Within the present study, we did only detect one transformation into small cell lung cancer as resistance mechanism to afatinib therapy and none in the population of first-generation EGFR TKI treated patients. Previous reports on smaller populations did not find transformation as a resistance mechanism to afatinib therapy [16, 17]. The detection of only one event in a cohort of 54 patients suggests that SCLC transformation is an even rarer event of resistance acquisition than in reversible *EGFR* TKI treated patients. Nevertheless identification of histological transformation, especially of resistant patients, that do not show any molecular mechanism, remains to be critical for treatment recommendations.

One re-biopsy sample showed an activating *CTNNB1* mutation, which is reported to confer resistance to *EGFR* therapies in non-small cell lung cancer [27]. No other acquired resistance mutations within the *BRAF*, *KRAS* or *PIK3CA* genes were identified in the present study. This could be either characteristic for afatinib treatment or due to the relatively small sample size. Nevertheless, two prospective studies on afatinib treated patients gathered the same observations [17, 22]. This is in contrast to first-generation *EGFR* TKI treatment, where *BRAF* and *PIK3CA* mutations account for 1 and 5% of resistance to treatment, respectively [28]. A review of Westover et al. [29] calculated KRAS mutation as a mechanism of EGFR TKI resistance to be at a frequency of approximately 1% from the majority of previously published data on resistance acquisition. Nevertheless, the underlying studies included all patients under EGFR TKI therapy without taking early progressors upon pre-existent resistance clones into account. Our study in contrast focused on acquired resistance, explicitly excluding early progressors. Only patients with a response to EGFR TKI therapy of at least 6 month were considered to be truly sensitive to therapy and did not carry any subclones with primary resistance mutations. This could be the reason for not identifying any KRAS mutation within the presented cohort of this study. Further, acquired resistance mechanisms via additional *EGFR* mutations (e.g. exon 20 duplication or p.D761Y and p.L747S) were not found either. This is in contrast to previous studies on first-generation EGFR TKIs, where up to 10% of patients under *EGFR* TKI therapy developed rare secondary mutations within the *EGFR* gene [30].

Activation of alternative pathways is the second common mechanism of resistance to EGFR TKIs. The emergence of anti-pan-HER treatment options (as afatinib) to block these alternative pathways by inhibiting phosphorylation of other HER family members was thought to solve this problem. Nevertheless, within this study, we examined downstream activation of the AKT pathway via amplification of the genes, encoding for *MET* and *ERBB2* to be equally abundant (2% vs 3%) in specific and pan-HER TKI treated patients. Downstream activation of the AKT pathway via amplification of the gene encoding the transmembrane kinase MET was shown to be the prominent alternative mechanism of resistance to first-generation EGFR TKI with 22% of cases [30]. Resistance acquisition by amplification of *ERBB2* is less common with a reported occurrence of 12% [30]*.* We here observed *MET* and *ERBB2* amplifications as exclusive mechanisms of resistance at lower frequencies than previously described. Additionally we found those in combination with other acquired resistance mechanisms like p.T790M or as a combination of *ERBB2* and *MET* amplification in the absence of a second-site mutation. The latter one is proposed to signal parallel to EGFR and thereby reactivates downstream signalling of the pathway [27].

Since, *EGFR* p.T790M is still the most common mechanism of resistance to afatinib, similar to reversible *EGFR* TKI, a comparison of patients´ prognosis and form of disease upon basis of the mutational profile should be made. Previously, patients were shown to have a better prognosis and more indolent form of disease progression upon the presence of an *EGFR* exon 19 deletion [31–34]. Matsuo and co-workers investigated whether there was an association of the (primary) *EGFR* driver mutation with the occurrence and frequency of *EGFR* p.T790M and the period of response to EGFR TKI. They observed a higher occurrence of *EGFR* p.T790M in patients with primary *EGFR* exon 19 deletions in contrast to *EGFR* p.L858R mutation (26 of 41 patients 63% vs 5 of 13 patients 38%) [35].

These findings are comparable to the prevalences observed within our cohort of first-generation EGFR TKI treatment, where primary EGFR exon 19 mutated patients developed a p.T790M more frequently than p.L858R mutated patients (74 and 54%). Interestingly, frequencies of p.T790M acquisition under afatinib therapy are comparable between primary *EGFR* exon 19 mutated patients and patients with a primary *EGFR* p.L858R mutation (44 and 45%). Matsuo and co-workers did not separate first-generation and second-generation TKIs and had only three afatinib patients in total, while in our study 55 afatinib patients were analysed. Therefore frequencies of co-occurrence of.primary EGFR mutation and p.T790M acquisition coincide with Matsuos´ observations for first-generation EGFR TKI but differ for afatinib. In our study, a clear numeric difference in the co-occurrence of primary driver mutation and frequency of p.T790M development can be seen in reversible EGFR TKI compared to afatinib-treated patients.

Treatment duration until progress under irreversible EGFR TKI in patients with or without acquired p.T790M revealed a comparable duration of 11 and 12 months. For p.T790M mutated patients, Tanaka et al. found a comparable time to progression of 12 months. In contrast, they observed that the duration of p.T790M negative patients under afatinib therapy was much lower with 4.5 months. This strong discrepancy can be explained by the differential study setting. Tanaka et al. analysed all patients under first-line afatinib therapy, while our study excluded early progressors (< 6 months until clinical progress). We further observed a prolonged PFS of patients under first-generation EGFR TKI, but this finding cannot be compared to results of of clinical trials (e.g.LUX-Lung 7, Archer1050) as our cohort included patients with a PFS > 6 months only. The differential study design to all published data on afatinib so far, is the fact, that patients with early progression (< 6 months) were excluded from this study. The data do not show the median PFS for the different generation of TKI for all patients treated, but only for the cohort with acquired resistance. The values are not comparable to median PFS from other studies, but rather should be seen as duration under therapy until resistance acquisition.

Molecular follow-up is of primary importance for second-line treatment decisions. As we could show within this study, the gatekeeper mutation p.T790M is the most prominent mechanism of resistance among all available first and second-generation EGFR TKI. This favours the majority of relapsed patients for third-generation EGFR TKI therapy (osimertinib), thereby prolonging overall survival. Nevertheless, the differential response of early and late progressors of first and second-generation EGFR TKI patients to osimertinib should be considered. Early progressors are characterized by the pre-existence of sub-clones carrying *EGFR* p.T790M that expand under EGFR TKI therapy. Those are supposed to better respond to osimertinib than late p.T790M resistant tumours that evolve from initially drug-tolerant cells [36, 37]. In conclusion, late progressors are the target population that profits more from first-line afatinib therapy followed by third-generation EGFR (osimertinib) therapy. In contrast, early progressors are supposed to profit from first-line osimertinib therapy [36, 38, 39]. Osimertinib, as probably the most effective way to prevent acquisition of the T790M resistance mutation, has been approved for first-line treatment in four countries including the US and Europe [40]. To stratify patients in the future, allele frequencies of *EGFR* p.T790M subclones in primary tumour samples should be evaluated via ultra-deep parallel sequencing [41–43]. Determination of a clinically relevant cut-off allele frequency could help to distinguish early and late progressors in advance. In future studies, a correlation of *EGFR* p.T790M allele frequency with therapeutic results of first-line afatinib treated patients should be performed.

Furthermore, the determination of tumor mutation burden with type and prevalence of resistance mutation acquisition could be of high interest in the future. Since TMB and efficacy of EGFR-Tyrosine kinase inhibitors in patients with EGFR-mutant lung cancers have been investigated and found to be negatively associated in lung cancer patient treated with EGFR TKI [44].

The present study is the first retrospective analysis based on a patient number as high as 123 first-line patients including 55 afatinib patients. Moreover, it considers the acquisition of resistance mutation under therapy by including only patients with at least 6 months response to EGFR TKI therapy. Furthermore, exclusively first-line treated tumours samples were considered for data evaluation. Therefore, this study avoids falsification of results by e.g. pre-existent resistance mutations per se or inclusions of patients with prior therapies.

## Conclusions

The *EGFR* p.T790M mutation is the most prominent mechanism of resistance to reversible and irreversible EGFR TKI therapy. Nevertheless there is a statistically significant difference of p.T790M acquisition between the two types of TKI, which might be of importance for clinical therapy decision.

## Supplementary information

**Additional file 1.**

## Data Availability

The datasets generated and analysed during the current study are not publicly available, but are available from the corresponding author on reasonable request.

## Abbreviations

AICDA: Activation-induced cytidine deaminase
ATP: Adenosintriphosphat
FISH: Fluorescence in situ hybridisation
NGS: Next generation sequencing
NSCLC: Non-small-cell lung cancer
PFS: Progression free survival
TKI: Tyrosine kinase inhibitor

## References

[1] Siegel R, Ma J, Zou Z, Jemal A (2014). Cancer statistics, 2014. CA Cancer J Clin.

[2] Maemondo M, Inoue A, Kobayashi K, Sugawara S, Oizumi S, Isobe H (2010). Gefitinib or chemotherapy for non-small-cell lung cancer with mutated EGFR. N Engl J Med.

[3] Gazdar AF (2009). Activating and resistance mutations of EGFR in non-small-cell lung cancer: role in clinical response to EGFR tyrosine kinase inhibitors. Oncogene..

[4] Köhler J, Schuler M (2013). Afatinib, Erlotinib and Gefitinib in the first-line therapy of EGFR mutation-positive lung adenocarcinoma: a review. Oncol Research and Treatment.

[5] Mok TS, Wu YL, Thongprasert S, Yang CH, Chu DT, Saijo N (2009). Gefitinib or carboplatin-paclitaxel in pulmonary adenocarcinoma. N Engl J Med.

[6] Mitsudomi T, Morita S, Yatabe Y, Negoro S, Okamoto I, Tsurutani J (2010). Gefitinib versus cisplatin plus docetaxel in patients with non-small-cell lung cancer harbouring mutations of the epidermal growth factor receptor (WJTOG3405): an open label, randomised phase 3 trial. Lancet Oncol.

[7] Janne PA, Yang JC, Kim DW, Planchard D, Ohe Y, Ramalingam SS (2015). AZD9291 in EGFR inhibitor-resistant non-small-cell lung cancer. N Engl J Med.

[8] Pao W, Miller VA, Politi KA, Riely GJ, Somwar R, Zakowski MF (2005). Acquired resistance of lung adenocarcinomas to gefitinib or erlotinib is associated with a second mutation in the EGFR kinase domain. PLoS Med.

[9] Nagano T, Tachihara M, Nishimura Y. Mechanism of Resistance to Epidermal Growth Factor Receptor-Tyrosine Kinase Inhibitors and a Potential Treatment Strategy. Cells. 2018;7(11):212. 10.3390/cells7110212 PMC6262543 30445769

[10] Takezawa K, Pirazzoli V, Arcila ME, Nebhan CA, Song X, de Stanchina E (2012). HER2 amplification: a potential mechanism of acquired resistance to EGFR inhibition in EGFR-mutant lung cancers that lack the second-site EGFRT790M mutation. Cancer Discov.

[11] Yamaoka T, Ohmori T, Ohba M, Arata S, Murata Y, Kusumoto S (2017). Distinct Afatinib resistance mechanisms identified in lung adenocarcinoma harboring an EGFR mutation. Mol Cancer Res.

[12] Shinohara S, Ichiki Y, Fukuichi Y, Honda Y, Kanayama M, Taira A (2018). Squamous cell carcinoma transformation from adenocarcinoma as an acquired resistance after the EGFR TKI therapy in (EGFR-mutated) non-small cell lung cancer. J Thorac Dis.

[13] Heigener DF, Schumann C, Sebastian M, Sadjadian P, Stehle I, Märten A (2015). Afatinib in non-small cell lung Cancer harboring uncommon EGFR mutations pretreated with reversible EGFR inhibitors. Oncologist.

[14] Liao BC, Lin CC, Yang JC (2015). Second and third-generation epidermal growth factor receptor tyrosine kinase inhibitors in advanced nonsmall cell lung cancer. Curr Opin Oncol.

[15] Yu HA, Arcila ME, Rekhtman N, Sima CS, Zakowski MF, Pao W (2013). Analysis of tumor specimens at the time of acquired resistance to EGFR-TKI therapy in 155 patients with EGFR-mutant lung cancers. Clin Cancer Res.

[16] Wu SG, Liu YN, Tsai MF, Chang YL, Yu CJ, Yang PC (2016). The mechanism of acquired resistance to irreversible EGFR tyrosine kinase inhibitor-afatinib in lung adenocarcinoma patients. Oncotarget..

[17] Tanaka K, Nosaki K, Otsubo K, Azuma K, Sakata S, Ouchi H (2017). Acquisition of the T790M resistance mutation during afatinib treatment in EGFR tyrosine kinase inhibitor–naïve patients with non–small cell lung cancer harboring EGFR mutations. Oncotarget..

[18] Wittersheim M, Heydt C, Hoffmann F, Buttner R (2017). KRAS mutation in papillary fibroelastoma: a true cardiac neoplasm?. J Pathol Clin Res.

[19] Konig K, Peifer M, Fassunke J, Ihle MA, Kunstlinger H, Heydt C (2015). Implementation of amplicon parallel sequencing leads to improvement of diagnosis and therapy of lung Cancer patients. J Thorac Oncol.

[20] Gultekin SE, Aziz R, Heydt C, Senguven B, Zoller J, Safi AF (2018). The landscape of genetic alterations in ameloblastomas relates to clinical features. Virchows Arch.

[21] Schildhaus HU, Schultheis AM, Ruschoff J, Binot E, Merkelbach-Bruse S, Fassunke J (2015). MET amplification status in therapy-naive adeno- and squamous cell carcinomas of the lung. Clin Cancer Res.

[22] Campo M, Gerber D, Gainor JF, Heist RS, Temel JS, Shaw AT (2016). Acquired resistance to first-line Afatinib and the challenges of prearranged progression biopsies. J Thorac Oncol.

[23] Remon J, Steuer CE, Ramalingam SS, Felip E (2018). Osimertinib and other third-generation EGFR TKI in EGFR-mutant NSCLC patients. Ann Oncol.

[24] Griesinger F, Radke S, Luers A, Deschler-Baier B, Kimmich M, Sebastian M (2018). Strategies to overcome acquired resistance to EGFR-TKI therapy based on T790M specific substances using Osimertinib as an example. Pneumologie..

[25] El Kadi N, Wang L, Davis A, Korkaya H, Cooke A, Vadnala V (2018). The EGFR T790M mutation is acquired through AICDA-mediated deamination of 5-methylcytosine following TKI treatment in lung cancer. Cancer Res.

[26] Sequist LV, Waltman BA, Dias-Santagata D, Digumarthy S, Turke AB, Fidias P (2011). Genotypic and histological evolution of lung cancers acquiring resistance to EGFR inhibitors. Sci Transl Med.

[27] Stewart EL, Tan SZ, Liu G, Tsao MS (2015). Known and putative mechanisms of resistance to EGFR targeted therapies in NSCLC patients with EGFR mutations-a review. Transl Lung Cancer Res.

[28] Ohashi K, Sequist LV, Arcila ME, Moran T, Chmielecki J, Lin YL (2012). Lung cancers with acquired resistance to EGFR inhibitors occasionally harbor BRAF gene mutations but lack mutations in KRAS, NRAS, or MEK1. Proc Natl Acad Sci U S A.

[29] Westover D, Zugazagoitia J, Cho BC, Lovly CM, Paz-Ares L (2018). Mechanisms of acquired resistance to first- and second-generation EGFR tyrosine kinase inhibitors. Ann Oncol.

[30] Morgillo F, Della Corte CM, Fasano M, Ciardiello F (2016). Mechanisms of resistance to EGFR-targeted drugs: lung cancer. ESMO Open.

[31] Oxnard GR, Arcila ME, Chmielecki J, Ladanyi M, Miller VA, Pao W (2011). New strategies in overcoming acquired resistance to epidermal growth factor receptor tyrosine kinase inhibitors in lung cancer. Clin Cancer Res.

[32] Oxnard GR, Janjigian YY, Arcila ME, Sima CS, Kass SL, Riely GJ (2011). Maintained sensitivity to EGFR tyrosine kinase inhibitors in EGFR-mutant lung cancer recurring after adjuvant erlotinib or gefitinib. Clin Cancer Res.

[33] Tian Y, Zhao J, Ren P, Wang B, Zhao C, Shi C, et al. Different subtypes of EGFR exon19 mutation can affect prognosis of patients with non-small cell lung adenocarcinoma. PLoS One. 2018;13(11):e0201682. 10.1371/journal.pone.0201682 PMC6211626 30383772

[34] Ricciuti B, Baglivo S, De Giglio A, Chiari R (2018). Afatinib in the first-line treatment of patients with non-small cell lung cancer: clinical evidence and experience. Ther Adv Respir Dis.

[35] Matsuo N, Azuma K, Sakai K, Hattori S, Kawahara A, Ishii H (2016). Association of EGFR exon 19 deletion and EGFR-TKI treatment duration with frequency of T790M mutation in EGFR-mutant lung Cancer patients. Sci Rep.

[36] Hata AN, Niederst MJ, Archibald HL, Gomez-Caraballo M, Siddiqui FM, Mulvey HE (2016). Tumor cells can follow distinct evolutionary paths to become resistant to epidermal growth factor receptor inhibition. Nat Med.

[37] Hochmair MJ, Morabito A, Hao D, Yang CT, Soo RA, Yang JC (2018). Sequential treatment with afatinib and osimertinib in patients with EGFR mutation-positive non-small-cell lung cancer: an observational study. Future Oncol.

[38] Attili I, Karachaliou N, Conte P, Bonanno L, Rosell R (2018). Therapeutic approaches for T790M mutation positive non-small-cell lung cancer. Expert Rev Anticancer Ther.

[39] Griesinger F, Roeper J (2018). Epidermal growth factor receptor tyrosine kinase inhibitors in advanced nonsmall cell lung cancer: what is the preferred first-line therapy?. Curr Opin Oncol.

[40] Osimertinib May Be an Effective First-Line Therapy in EGFR-Mutant NSCLC (2017). Cancer Discovery.

[41] Mao X, Zhang Z, Zheng X, Xie F, Duan F, Jiang L (2017). Capture-based targeted Ultradeep sequencing in paired tissue and plasma samples demonstrates differential subclonal ctDNA-releasing capability in advanced lung Cancer. J Thorac Oncol.

[42] Campbell PJ, Pleasance ED, Stephens PJ, Dicks E, Rance R, Goodhead I (2008). Subclonal phylogenetic structures in cancer revealed by ultra-deep sequencing. Proc Natl Acad Sci U S A.

[43] Kohsaka S, Petronczki M, Solca F, Maemondo M (2018). Tumor clonality and resistance mechanisms in EGFR mutation-positive non-small-cell lung cancer: implications for therapeutic sequencing. Future Oncol.

[44] Offin M, Rizvi H, Tenet M, Ni A, Sanchez-Vega F, Li BT (2018). Tumor mutation burden and efficacy of EGFR-tyrosine kinase inhibitors in patients with EGFR-mutant lung cancers. Clin Cancer Res.

